# A Bibliometric Analysis of the 20 Most Cited Articles on Sacrococcygeal Chordomas

**DOI:** 10.7759/cureus.61119

**Published:** 2024-05-26

**Authors:** Apratim Maity, Max Ward, Ethan D Brown, Daniel M Sciubba, Sheng-Fu L Lo

**Affiliations:** 1 Neurological Surgery, Donald and Barbara Zucker School of Medicine at Hofstra/Northwell, Manhasset, USA

**Keywords:** spine, sacrococcygeal chordomas, sacral chordomas, coccyx chordomas, coccygeal chordomas, bibliometric

## Abstract

This study aims to summarize sacrococcygeal chordoma literature through bibliometric analysis and to offer insights into key studies to guide clinical practices and future research. The Web of Science database was searched using the terms “sacral chordoma”, “chordomas of the sacrum”, “chordomas of the sacral spine”, “chordomas of the sacrococcygeal region”, “coccygeal chordoma”, and “coccyx chordoma". Articles were analyzed for citation count, authorship, publication date, journal, research area tags, impact factor, and evidence level. The median number of citations was 75 (range: 53-306). The primary publication venue was the International Journal of Radiation Oncology, Biology, Physics. Most works, published between 1999 and 2019, featured a median journal impact factor of 3.8 (range: 2.1-7) and predominantly fell under the research area tag, radiation, nuclear medicine, and imaging. Of these articles, 19 provided clinical data with predominantly level III evidence, and one was a literature review. This review highlights the increasing volume of sacrococcygeal chordoma publications over the past two decades, indicating evolving treatment methods and interdisciplinary patient care. Advances in radiation, particularly intensity-modulated radiation therapy (IMRT) and proton beam therapy, are believed to be propelling research growth, and the lack of level I evidence underscores the need for more rigorous studies to refine treatment protocols for sacrococcygeal chordomas.

## Introduction and background

Chordomas are low-grade, slow-growing tumors of the sarcoma family, which arise from remnants of the notochord [1]. The notochord is an embryonic mesodermal structure that marks the midline of the embryo and induces the formation of the neural tube. The neural tube develops into the central nervous system, while the notochord develops into the nucleus pulposus, located within the intervertebral discs of the vertebral column. In adults, remnants of the primitive notochord may remain within the skull base or spinal column. Neoplastic growths within these notochordal remnants are referred to as chordomas. Sacrococcygeal chordomas make up 50% of all chordomas [2], and due to their slow growth rate and nonspecific presentation, they tend to be diagnosed late [3]. By the time treatment is initiated, most sacrococcygeal chordomas have advanced significantly. A high incidence of recurrence and diminished five-year (67.6%) and 10-year (39.9%) survival rates contribute to these tumors having a poor prognosis [1,4].

The significant mortality associated with sacral chordomas makes identifying significant and impactful contributions within the existing literature of clinical interest. Bibliometric analysis is a research method that allows for the mapping of the scientific landscape, reveals emerging trends and shifts within the research focus, and helps in conducting comprehensive literature reviews while displaying gaps in the existing body of knowledge for a specific field or topic [5]. While bibliometric analysis of chordomas has already been performed, the present study represents the first bibliometric analysis focusing specifically on chordomas of the sacrum and coccyx, which is its most common presentation within the neuraxis. This study provides clinicians and researchers with a brief overview of the literature on sacrococcygeal chordomas and identifies key characteristics of the most cited research articles.

## Review

Methods

To determine the 20 most cited articles on sacral chordomas, the Web of Science research database and its citation index services were used. A filtered search was conducted for articles that contained the phrases “sacral chordoma”, “chordomas of the sacrum”, “chordomas of the sacral spine”, “chordomas of the sacrococcygeal region”, "coccygeal chordoma”, or “coccyx chordoma” within either the title or keywords. No filter related to the publication year of articles was set. The results were then sorted by the highest number of citations to the lowest number of citations. Using the export function within the Web of Science database, the search results were exported into Microsoft Excel, and the articles were organized for further analysis. Similar to the methodology found in other bibliometric analyses, each article was analyzed for its number of citations, first author, year of publication, journal of publication, research area tags, journal impact factor, and level of evidence [6]. Research area tags are tags applied by the Web of Science to research articles within their database. Articles may contain tags for multiple disciplines (e.g. “clinical neurology” and “orthopedics”). All of the aforementioned data, except for journal impact factor and level of evidence, was obtained using the Web of Science database. Journal impact factors were collected from each journal’s web page, and for clinical articles, the level of evidence was determined using the Oxford Centre for Evidence-based Medicine categorization system. Literature reviews were omitted from the clinical article analysis. The categories were randomized controlled trials (RCTs) (level I evidence), nonrandomized controlled or prospective cohort trials (level II evidence), retrospective cohort studies (level III evidence), case series (level IV evidence), and expert opinion or observational articles (level V evidence). All statistical data analyses were conducted using Microsoft Excel.

Results

The 20 most cited articles on sacral chordomas (Table 1) contained a total of 2,046 combined citations, with a median number of citations at 75 and a range between 53 and 306 citations. The oldest article was published in 1999, while the newest was published in 2019 (Table 2). The journal in which the most articles were published was the International Journal of Radiation Oncology, Biology, Physics (n = 4), followed by the British Journal of Radiology (n = 2), Journal of Bone and Joint Surgery-America Volume (n = 2), Neurosurgery (n = 2), and Spine (Phila Pa 1976) (n = 2), with the second most cited articles (Table 3). The average journal impact factor for these articles was 4.05, with a median impact factor of 3.8 and a range between 2.1 and 7. The average impact factor for the top 5 articles was 5.13, while the average impact factor for the bottom 15 articles was 4.38. However, a two-sample t-test showed that this difference was not significant (p = 0.421). The most common research area tag within the top 20 articles was radiation, nuclear medicine, and medical imaging (n = 8), followed by orthopedics (n = 7), surgery (n = 7), oncology (n = 7), clinical neurology (n = 6), and rehabilitation (n = 1). One article was a literature review, while the remaining 19 articles presented clinically obtained information. Out of these, the majority provided level III evidence (n = 17) (Table 4).

**Table 1:** Top 20 Cited Articles

Rank	First Author	Title	Journal	Citations	Journal Impact Factor
1	Fuchs [7]	Operative management of sacral chordoma	Journal of Bone and Joint Surgery-American Volume	306	4.578
2	York [8]	Sacral chordoma: 40-year experience at a major cancer center	Neurosurgery	287	5.315
3	Hulen [9]	Oncologic and functional outcome following sacrectomy for sacral chordoma	Journal of Bone and Joint Surgery-American Volume	154	4.578
4	Hsieh [10]	Long-term clinical outcomes following en bloc resections for sacral chordomas and chondrosarcomas: a series of twenty consecutive patients	Spine (Phila Pa 1976)	138	4.166
5	Hof [11]	Effectiveness of cetuximab/gefitinib in the therapy of a sacral chordoma	Onkologie	118	7
6	Imai [12]	Carbon ion radiation therapy for unresectable sacral chordoma: an analysis of 188 cases	International Journal of Radiation Oncology Biology Physics	113	2.4
7	Hanna [13]	Sacral chordoma: can local recurrence after sacrectomy be predicted?	Clinical Orthopaedics and Related Research	95	4.837
8	Varga [14]	Surgical treatment of sacral chordoma: prognostic variables for local recurrence and overall survival	European Spine Journal	81	3.2
9	Yin [15]	Comparison of radiomics machine-learning classifiers and feature selection for differentiation of sacral chordoma and sacral giant cell tumour based on 3d computed tomography features	European Radiology	79	5.9
10	Kayani [16]	A review of the surgical management of sacral chordoma	European Journal of Surgical Oncology	78	3.8
11	Imai [17]	Carbon ion radiotherapy for sacral chordoma	British Journal of Radiology	72	3.639
12	Chen [18]	Prognostic factors of sacral chordoma after surgical therapy: a study of 36 patients	Spinal Cord	67	2.2
13	Imai [19]	Effect of carbon ion radiotherapy for sacral chordoma: results of phase I-II and phase II clinical trials	International Journal of Radiation Oncology Biology Physics	63	7
14	Nishida [20]	Clinical outcome of sacral chordoma with carbon ion radiotherapy compared with surgery	International Journal of Radiation Oncology Biology Physics	61	7
15	Kabolizadeh [21]	Updated outcome and analysis of tumor response in mobile spine and sacral chordoma treated with definitive high-dose photon/proton radiation therapy	International Journal of Radiation Oncology Biology Physics	58	7
16	Mima [22]	Particle therapy using carbon ions or protons as a definitive therapy for patients with primary sacral chordoma	British Journal of Radiology	58	4.166
17	Radaeilli [23]	Sacral chordoma: long-term outcome of a large series of patients surgically treated at two reference centers	Spine (Phila Pa 1976)	57	3.639
18	Smolders [24]	Value of MRI in the diagnosis of non-clival, non-sacral chordoma	Neurosurgery	55	5.315
19	Yamada [25]	Preliminary results of high-dose single-fraction radiotherapy for the management of chordomas of the spine and sacrum	Journal of Surgical Oncology	53	3.454
20	Angelini [26]	Prognostic factors in surgical resection of sacral chordoma	Journal of Bone and Joint Surgery-American Volume	53	4.578

**Table 2:** Time Period That Generated the Most Cited Articles

Decade	Number of Articles
1999-2009	7
2010-2020	13

**Table 3:** Journals With the Most Cited Articles

Journal	Number of Articles
International Journal of Radiation Oncology, Biology, Physics	4
British Journal of Radiology	2
Journal of Bone and Joint Surgery. American Volume	2
Neurosurgery	2
Spine (Phila Pa 1976)	2
Clinical Orthopaedics and Related Research	1
European Journal of Surgical Oncology	1
European Radiology	1
European Spine Journal	1
Journal of Surgical Oncology	1
Oncology Research and Treatment	1
Skeletal Radiology	1
Spinal Cord	1

**Table 4:** Evidence Level for Clinical Studies

Evidence Level	Number of Articles
I	0
II	0
III	17
IV	2
V	0

Discussion

Analysis of the Top 3 Most Cited Articles

While bibliometric analyses covering chordomas have been conducted, this is the first bibliometric analysis focusing exclusively on sacrococcygeal chordomas. The articles identified within this study highlight some of the most influential contributions to sacral chordomas, and while each of these articles deserves lengthy discussion, we begin by providing an overview of the top 3 most cited articles.

The most cited article (n = 306) by Fuchs et al. was a retrospective cohort study comparing the efficacy of various surgical approaches, including the posterior approach in 22 patients and the anteroposterior approach in 30 patients, in achieving a wide surgical margin, as well as the association of a wide surgical margin with patient outcomes [7]. Broadly, they observed a recurrence-free rate of survival of 74% at five years, 52% at 10 years, and 47% at 15 years. The investigators reported that of 21 patients who achieved a wide surgical margin, 17 had undergone an anteroposterior approach vs four who had undergone a posterior approach. A wide margin was highly predictive of patient outcomes, with 20/21 patients who achieved a wide margin demonstrating recurrence-free survival during the 21-year observation window vs 9/31 who did not achieve a wide margin. Additionally, the authors noted no relationship between radiation treatment and either survival or disease-free status. However, they acknowledged limitations in making this conclusion, as less than half of investigated patients utilized radiation and two-thirds of those undergoing radiotherapy received it for reoccurrence. Ultimately, the authors concluded that a wide surgical margin was the most important predictor of patient outcome in surgically treated sacral chordomas and that the use of an anteroposterior approach yielded the greatest chance of achieving a wide margin.

The second most cited article (n = 287) by York et al. was a retrospective cohort study done to evaluate the effects of various treatment modalities, including radiotherapy and surgical excision, on the course of sacral chordoma disease [27]. Within their study, the investigators assessed the Kaplan-Meier survival time, tumor recurrence, and disease-free interval of 27 sacral chordoma patients. All investigated patients underwent at least one surgical procedure, and 15/27 underwent two or more operations. High levels of disease reoccurrence were observed, with 47/67 procedures yielding tumor reoccurrence. The investigators identified a large difference in a disease-free interval for patients who underwent radical resection rather than sub-total excision (2.27 years vs 8 months). Additionally, they observed a disease-free interval prolonging effect of radiotherapy for patients with only subtotal resection (2.12 years vs eight months). However, they acknowledged the limited usefulness of this finding, as radiation therapy would be contraindicated for patients undergoing multiple surgical excisions. The investigators concluded that, given the high rates of chordoma reoccurrence and disparities in the disease-free interval, radical resection should be the treatment of choice for sacral chordoma, and radiotherapy should be considered, whenever possible, following sub-total resection.

The third most cited article (n = 154) by Hulen et al. was a retrospective cohort study that aimed to investigate the oncologic and functional outcomes of patients undergoing sacrectomy for sacral chordoma [28]. The investigators assessed recovery from the initial surgical intervention in 16 patients using recurrence rate, survival, functional outcome measures, and rate of complications. Tumor recurrence was observed in 12/16 patients, disease-free status in 4/16 patients at a mean of 94.5 months, and death from disease in 5/16 patients at a mean of 31.4 months. Additionally, 15/16 patients failed to regain normal bowel and bladder post-operatively, 13/16 were unable to walk without assistive devices, and 8/16 demonstrated post-operative wound complications. Greater levels of cephalad resection were associated with worse bowel and bladder control. No association between radiotherapy or negative margins and survival or local reoccurrence was observed. The investigators suggested that a limited sample size prevented them from demonstrating a significant effect of omental flap usage on complication incidence and that functional outcomes described in their article were limited to those effecting bowel, bladder, and walking status. The investigators concluded that complete resection and aggressive treatment of local reoccurrence should be pursued when possible.

Key Findings From the 20 Most Cited Articles

Table 5 lists the key findings from the 20 most cited articles on sacral chordomas. The findings are listed in order of rank from most cited article to least cited article.

**Table 5:** Key Findings From the 20 Most Cited Articles

Rank	Authors	Key Findings
1	Fuchs et al. [7]	Showed that a wide surgical margin is the most important predictor of survival and of local recurrence in patients with sacrococcygeal chordomas.
2	York et al. [8]	Showed that radical resection is associated with a significantly longer disease-free interval, compared with subtotal removal of the tumor. Authors suggest that radical resection should be the treatment of choice for sacral chordomas.
3	Hulen et al. [9]	Showed that 1) treating sacral chordomas requires a multidisciplinary approach, 2) frequent recurrence and late onset of metastatic disease are to be expected in a substantial proportion of sacral chordoma patients, and 3) surgical treatment often results in substantial functional impairment and numerous complications.
4	Hsieh et al. [10]	Showed that wide or marginal en bloc excision of sacral chordoma and chondrosarcoma is associated with significant improvement in disease-free survival with acceptable perioperative morbidity rate.
5	Hof et al. [11]	Showed that the inhibition of the EGF pathway may be an effective measure in the treatment of a chordoma. Authors suggest that further follow-up will have to done to prove its long-term efficiency.
6	Imai et al. [12]	Showed that carbon ion radiation therapy is safe and effective for unresectable chordoma and provides good local control and survival while preserving ambulation.
7	Hanna et al. [13]	Showed that obtaining wide surgical margins posteriorly, by excising parts of the piriformis, gluteus maximus, and sacroiliac joints, may result in better local disease control in patients with sacral chordoma.
8	Varga et al. [14]	Identified two predictive variables for local recurrence-free survival (previous tumor surgery and type of surgical resection) and two for overall survival (age and impaired motor function) in surgically treated SC patients. Their results also indicate that en bloc resection reduces local recurrence but does not influence overall survival; authors do suggest that this finding is likely due to a short follow-up period.
9	Yin et al. [15]	Showed that 1) sacral chordoma and sacral giant cell tumor are the two most common primary tumors of the sacrum with many common clinical and imaging characteristics, 2) a radiomics model helps clinicians to identify the histology of a sacral tumour, and 3) CT-enhanced features should be preferred over non-enhanced CT features when using machine-learning methods to preoperatively differentiate between sacral chordomas and sacral giant cell tumors.
10	Kayani et al. [16]	Showed that 1) operative resection with wide resection margins offers the best long-term prognosis, 2) Inadequate resection margins, large tumor size, dedifferentiation, and greater cephalad chordoma extension are associated with poor oncological outcomes, and 3) routine long-term follow-up is essential to enable early detection and treatment of recurrent disease.
11	Imai et al. [17]	Showed that carbon ion radiotherapy appears to be effective and safe in the management of patients with sacral chordoma and offers a promising alternative to surgery.
12	Chen et al. [18]	Showed that 1) higher level of tumor involvement, invasion into the surrounding muscle, incomplete excision, and inadequate surgical margins are poor prognostic factors for patients with sacral chordomas, and 2) resecting the tumor completely with wide surgical margins may provide a better prognosis for these patients.
13	Imai et al. [19]	Showed that carbon ion radiotherapy appears to be effective and safe in the management of patients with sacral chordoma and offers a promising alternative to surgery.
14	Nishida et al. [20]	Showed that carbon ion radiotherapy results in a high local control rate and preservation of urinary-anorectal function compared with surgery.
15	Kabolizadeh et al. [21]	Showed that 1) the use of high-dose definitive radiation therapy for selected patients with unresected spine and sacral chordomas is effective, 2) assessment of tumor response to radiation therapy by volumetric analysis is superior to modified RECIST analysis in chordoma patients, and 3) evaluating the soft tissue target volume is an excellent indicator of tumor response.
16	Mima et al. [22]	Showed that 1) particle therapy for patients with sacral chordomas showed favorable local recurrence and overall survival, 2) severe toxicities were successfully reduced by modifying the dose fractionation and treatment planning in the later treatment era. Authors conclude that particle therapy should be considered useful and safe for patients with sacral chordomas.
17	Radaelli et al. [23]	Showed that 1) the long-term outcome of resected sacral chordomas was poor, with less than 25% of patients achieving disease-free status at 15 years, and 2) when surgical margins are expected to be positive, other treatment modalities should be considered, especially when expected sequelae are substantial as in the case of more cephalad levels of resection.
18	Smolders et al. [24]	Showed that although the signal intensity on MR imaging is not specific, chordoma should be considered when a destructive lesion of a vertebral body is associated with a soft tissue mass that has a collar button or mushroom appearance and dumbbell morphology and spans several vertebral segments while sparing the disk(s).
19	Yamada et al. [25]	Showed that high-dose single-fraction stereotactic radiosurgery provides good tumor control with low treatment-related morbidity. Authors suggest that additional follow-up, however, is required to determine the long-term recurrence risk.
20	Angelini et al. [26]	Showed that 1) the most prominent adverse factor for local recurrence was previous intralesional surgery, and 2) local recurrence rate was related with inadequate surgical margins and tumor volume.

Trends in Sacral Chordoma Research

The International Journal of Radiation Oncology, Biology, Physics was shown to be the predominant journal in which research pertaining to sacral chordomas was published, followed by journals with focuses on radiology, orthopedics, neurosurgery, and surgical oncology. Furthermore, when analyzing the departments of the first authors of these articles, we found that most are within the orthopedics department (n = 8), followed by radiation oncology (n = 5), radiology (n = 2), neurosurgery (n = 2), and surgery (n=1) departments. This distribution of journals and first-author departments highlights the interdisciplinary nature of sacral chordoma research, which encompasses a wide range of clinical and therapeutic specialties.

Broadly speaking, the subject matter of sacral chordoma research appears to be focused on three areas: radiation therapy, understanding the genetic basis of disease, and surgical management. Additionally, as evidenced in Table 4, the majority of articles provided level III evidence (n = 17 out of 19 clinical articles) likely due to the low incidence of chordomas limiting randomization opportunities. One literature review was documented pertaining to the prognostic factors and optimal treatment modality of sacral chordoma [16], but this review was omitted from the evidence analysis. No articles among the top 20 most cited were classified as translational research.

Most articles focus on comparing the outcomes of surgery versus radiation. Between 1990 and 2009, a 19-year time span, seven of the most cited articles were published on the topic of sacral chordomas compared to 13 between 2010 and 2019. It is possible that the increased referencing of these newer articles largely correlates with significant improvements in radiation therapy techniques after 2010, specifically techniques such as intensity-modulated radiation therapy (IMRT), stereotactic body radiotherapy (SRBT), proton beam therapy, and image-guided radiation therapy (IGRT) [29]. Proton beam therapy is considered the best radiation option for chordomas as proton therapy minimizes the exposure of healthy tissue to high-dose radiation [30]. According to the American Cancer Society, the use of proton beam therapy for treating all types of cancer showed the greatest increase after 2010 (from 0.4% to 2.2%) [29]. It is likely that we will see a continued rise in radiation research as the techniques and technologies improve even further.

We do acknowledge two limitations of our bibliometric analysis. The first limitation is that articles discussing sacral chordomas, but do not mention them in their title or keywords, as described within our search terms, may exist and are not represented within the data. However, these articles are likely to only mention sacral chordomas briefly rather than as a point of focus. Secondly, this study did not account for articles on “chordomas” that also mention “sacral chordomas” because data on these articles exist within other bibliometric analyses, such as that conducted by Ikpeze et al. [30].

## Conclusions

Bibliometric analysis is a tool used to evaluate the existing research landscape of a particular topic. In this study, we have highlighted impactful contributions that can guide future research and assist in the treatment of sacral chordomas and articles that made them all more important because of the relatively high prevalence of sacral chordomas within the category of brain and spine tumors. Our bibliometric analysis has shown a discernible increase in published literature throughout the last decade, reflecting an evolution in treatment modalities and a multidisciplinary approach to patient care. While advancements in radiation techniques, such as IMRT and proton beam therapy, are likely driving the uptick in research, the large amounts of studies citing level III evidence within the most cited articles demonstrate a need for more rigorous studies to refine treatment protocols further. Level III evidence studies such as retrospective cohort studies, case-control studies, and case series studies are more prone to bias and confounding variables in comparison to level I or II evidence studies, such as randomized controlled trials or non-randomized controlled trials, respectively. However, overcoming the practical and ethical barriers to conducting these types of studies in rare disease research continues to pose a challenge for future research to come.
